# Dietary Genistein Could Modulate Hypothalamic Circadian Entrainment, Reduce Body Weight, and Improve Glucose and Lipid Metabolism in Female Mice

**DOI:** 10.1155/2019/2163838

**Published:** 2019-04-17

**Authors:** Liyuan Zhou, Xinhua Xiao, Qian Zhang, Jia Zheng, Ming Li, Miao Yu, Xiaojing Wang, Mingqun Deng, Xiao Zhai, Rongrong Li, Jieying Liu

**Affiliations:** Key Laboratory of Endocrinology, Translational Medicine Center, Ministry of Health, Department of Endocrinology, Peking Union Medical College Hospital, Peking Union Medical College, Chinese Academy of Medical Sciences, Beijing, China

## Abstract

Genistein has beneficial effects on metabolic disorders. However, the specific mechanism is not clearly understood. In light of the significant role of the hypothalamus in energy and metabolic homeostasis, this study was designed to explore whether dietary genistein intake could mitigate the harmful effects of a high-fat diet on glucose and lipid metabolism and whether any alterations caused by dietary genistein were associated with hypothalamic gene expression profiles. C57BL/6 female mice were fed a high-fat diet without genistein (HF), a high-fat diet with genistein (HFG), or a normal control diet (CON) for 8 weeks. Body weight and energy intake were assessed. At the end of the study, glucose tolerance and serum levels of insulin and lipids were analyzed. Hypothalamic tissue was collected for whole transcriptome sequencing and reverse transcription quantitative PCR (RT-qPCR) validation. Energy intake and body weight were significantly reduced in the mice of the HFG group compared with those of the HF group. Mice fed the HFG diet had improved glucose tolerance and decreased serum triacylglycerol, free fatty acids, and low-density lipoprotein cholesterol compared with those fed the HF diet. The HFG diet also modulated gene expression in the hypothalamus; the most abundant genes were enriched in the circadian entrainment pathway. Dietary genistein intake could reduce body weight, improve glucose and lipid metabolism, and regulate hypothalamic circadian entrainment. The ability of genistein intake to influence regulation of the hypothalamic circadian rhythm is important since this could provide a novel target for the treatment of obesity and diabetes.

## 1. Introduction

The International Diabetes Federation (IDF) newly announced that there were more than 425 million adults living with diabetes worldwide in 2017. If this growing trend continues, the number of individuals with diabetes will rise to 629 million in 2045[1]. The epidemic of diabetes has brought unprecedented challenges to public health and the economy. Type 2 diabetes mellitus (T2DM) accounts for almost 90% of the diabetes cases found in the diabetic population [2–4]. Overweight and obesity have been considered independent risk factors for T2DM. Thus, great action is urgently required to improve glucose metabolism, regulate body weight, and reduce the global burden of T2DM and obesity.

In recent years, the effects of dietary bioactive components on chronic diseases have been widely explored [5]. Several large epidemiological studies [6–8] have demonstrated that soy isoflavone intakes are negatively associated with the development of T2DM. Genistein, one of the major components of soy isoflavone, is a member of the phytoestrogen family, resulting from its analogous structure with 17*β*-estradiol, and is extensively present in the infant formula in China. It has been reported that genistein has protective effects against cancer and cardiovascular diseases [9]. Furthermore, a growing number of clinical trials [10–13] and animal experiments [14, 15] indicated that genistein intake could ameliorate glucose intolerance, insulin resistance, serum lipid disturbances, and nonalcoholic fatty liver disease and regulate weight. In addition to the promotion of *β*-cell proliferation and inhibition of apoptosis, estrogenic effects and tyrosine kinase inhibitors were also considered potential antidiabetes mechanisms of genistein [9]; however, the specific mechanisms of genistein improving glucose and lipid metabolism are still incompletely understood.

The hypothalamus is the center of energy regulation and glucose homeostasis, and it could sense and integrate metabolic signals from peripheral metabolic organs, such as the pancreas, liver, fat, and muscle, to maintain energy and glucose homeostasis. There were corresponding nuclei in the hypothalamus for controlling various aspects of homeostatic regulation of energy metabolism. During the last few decades, central regulation of glucose metabolism has received extensive attention in academia. A large number of studies have discovered that central regulation disorders are one of the most important pathogeneses of obesity and T2DM [16, 17]. In addition, circadian rhythm has a key role in determining physical homeostasis and metabolic health. The hypothalamus is also the central clock for regulating circadian rhythm and is crucial in coordinating the light-dark cycle with metabolism in peripheral tissues. The generation and control of circadian rhythms in the hypothalamus are also potential mechanisms for maintaining energy and metabolic homeostasis. Recently, emerging studies showed that circadian rhythm disorders played significant roles in obesity [18, 19] and metabolic disturbances [20–22]. Although the beneficial effects of genistein on cognitive function [23] and regulation of hormone levels by the hypothalamus [24–26] have been explored, investigations into the central regulation mechanisms of genistein intervention on glucose and lipid metabolism and body weight are limited.

The present study was designed to explore the effects of dietary genistein on glucose and lipid metabolism and body weight and discuss whether genistein intake can mitigate the deleterious metabolic influences of a high-fat diet. In addition, the central mechanisms by which dietary genistein regulates metabolism were determined using hypothalamic whole transcriptome sequences.

## 2. Materials and Methods

### 2.1. Animals and Study Design

Four-week-old C57BL/6J female mice were purchased from the National Institutes for Food and Drug Control (Beijing, China; SCXK-2014-0013). The mice were housed under SPF (specific pathogen-free) conditions (room temperature of 20-24°C and 12 h light/night cycles) with ad libitum access to water and a normal control diet (AIN-93G, Research Diets, USA). After one week of adaptation, the mice were randomly assigned to three groups and were fed either a high-fat diet without genistein (HF, n=8), a high-fat diet with genistein (CAS: 466-72-0, G0272, TCI Development Co., Ltd) (2 g/kg diet) (HFG, n=8), or a normal control diet (CON, n=8). Soybean oil was substituted with corn oil in both the high-fat diet and the normal control diet. The nutrient composition is shown in supplementary Table S1. The HF diet included 60% of calories from fat and supplied 5.24 kcal/g energy, whereas the CON diet contained 15.8% of calories derived from fat and supplied 3.9 kcal/g energy. After eight weeks of genistein treatment, blood samples were collected from the intraorbital retrobulbar plexus after 10 h of fasting. The hypothalamus was removed as previously described [27] and then stored at -80°C for further analysis. All operations were conducted under chloral hydrate anesthesia, and the best efforts were made to minimize suffering. All of the procedures were approved by the animal care and use committee of the Peking Union Medical College Hospital (Beijing, China, SYXK-2018-0019). All of the animal operations were conducted in compliance with the Guide for the Care and Use of Laboratory Animals.

### 2.2. Measurement of Body Weight, Food Intake, and Energy Intake

Body weight and food intake were measured once per week; the food intake was estimated by weighing the remaining food. Energy intake was assessed by multiplying the food intake by the energy supply of the diet.

### 2.3. Glucose Tolerance Test (GTT)

At the end of treatment, an intraperitoneal glucose tolerance test (IPGTT) was performed. After fasting for 6 h, the mice were injected intraperitoneally with a glucose load of 2 g/kg body weight. The blood glucose levels were measured in the tail vein before (0 min) and at 30, 60, and 120 min after the injection using a Contour TS Glucometer (ACCU-CHEK Mobile, Beijing, China). In addition, the area under the curve (AUC) of the IPGTT results was calculated [28].

### 2.4. Measurement of Biochemical Parameters

Blood samples were collected after eight weeks of intervention and centrifuged at 3000×g for 10 min at 4°C. The serum total cholesterol (TC), triglycerides (TG), low-density lipoprotein cholesterol (LDL-C), high-density lipoprotein cholesterol (HDL-C), and free fatty acids (FFA) were measured by routine automated laboratory methods. Fasting insulin levels were detected using a mouse insulin ELISA Kit (80-INSMSU-E01, Salem, NH, USA). The homeostasis model assessment of insulin resistance (HOMA-IR) was used to assess insulin sensitivity. The calculation of the HOMA-IR was the same as previously described [28].

### 2.5. RNA Preparation and Whole Transcriptome Sequencing

The gene expression levels in the hypothalamic tissues were detected using whole transcriptome sequencing analyses (n=3 per group). TRIzol reagent (Life Technologies Inc., Carlsbad, CA, USA) was used to extract total RNA from the hypothalamic tissues. The degradation and contamination of RNA was monitored on 1% agarose gels. RNA concentration was measured using a Qubit RNA Assay Kit in a Qubit 2.0 Fluorometer (Life Technologies, CA, USA). All RNA samples had high quality and were without degradation and contamination. Two micrograms of RNA per sample were used as input material for the RNA library preparations. A NEBNext UltraTM RNA Library Prep Kit from Illumina (NEB, USA) was used to generate sequencing libraries, and index codes were added to attribute sequences to each sample. Clustering of the index-coded samples was performed on a cBot Cluster Generation System using the TruSeq PE Cluster Kit v4-cBot-HS (Illumina) according to the manufacturer's instructions. After cluster generation, the library preparations were sequenced on an Illumina HiSeq 4000 platform, and paired-end 150 bp reads were generated.

After quality control, the clean reads were mapped to the reference genome sequence (Mus musculus (assembly GRCm38.p6), NCBI) using Tophat2 tools software. Differential expression analysis of the two groups was performed using the DESeq R package (1.10.1). P-values < 0.05 found by DESeq were considered differentially expressed. To discover the biological significance of the altered genes, the enrichment of differentially expressed genes in KEGG (Kyoto Encyclopedia of Genes and Genomes) pathways was analyzed using KOBAS software [29].

### 2.6. Reverse Transcription Quantitative PCR (RT-qPCR) Experiments

To validate the results of whole transcriptome sequencing, we selected three genes from the gene list for RT-qPCR analysis (n=5 per group). Total RNA was prepared as mentioned above. Then, 1.0 *μ*g of total RNA was reverse transcribed into cDNA using the PrimeScript™ RT Reagent Kit with gDNA Eraser (RR047A, TaKaRa Bio Inc., Otsu, Shiga, Japan). cDNA (2 *μ*l) was amplified on an ABI 7500 thermocycler (Applied Biosystems, CA, USA) using the TB Green PCR Master Mix (RR820A, Takara Bio Inc., Otsu, Shiga, Japan) in a total volume of 20 *μ*l. The reaction conditions included an initial denaturation step (30 s at 95°C) and a cycling step (denaturation for 5 s at 95°C and annealing and extending for 34 s at 60°C for 40 cycles). *β*-actin was used for normalization. All the sequences of the primers are listed in Table S2 in the supplementary material. The relative expression levels of the genes were quantified by the 2^−△△Ct^ method.

### 2.7. Statistical Analysis

The data are expressed as the mean ± standard error of the mean (S.E.M). The statistics of multiple comparisons were analyzed by one-way ANOVA and two-way ANOVA with Tukey and Bonferroni post-hoc analyses. Student's t-test was used to compare unpaired samples. A* p *value < 0.05 was considered statistically significant. Prism version 7.0 (GraphPad Software Inc., San Diego, CA, USA) was used for statistical analysis.

## 3. Results

### 3.1. Dietary Genistein Reduces Body Weight and Energy Intake

**Figure 1:**
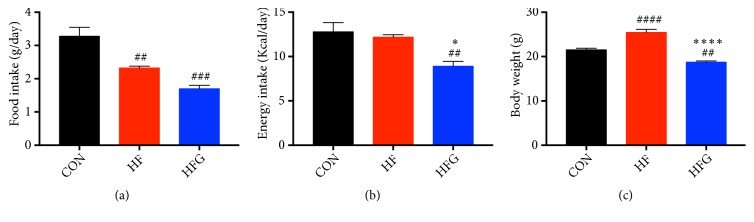
Dietary genistein could reduce energy intake and body weight in mice fed a high-fat diet. (a) Mean food intake during the experimental period expressed as grams per day; (b) mean energy intake expressed as kcal per day; (c) body weight by the end of the study. CON: normal control diet; HF: high-fat diet without genistein; HFG: high-fat diet with genistein. Data are expressed as means ± S.E.M. (n=8/group). Mean values were significantly different between other groups and the CON group: ^#^*p*<0.05; ^##^*p*<0.01; ^###^*p*<0.001; ^####^*p*<0.0001. Mean values were significantly different between HF group and the HFG group: ^*∗*^*p*<0.05, ^*∗∗*^*p*<0.01, ^*∗∗∗*^*p*<0.001, ^*∗∗∗∗*^*p* <0.0001.

During the eight-week genistein intervention, the food intake of mice in the HF group (*p*<0.01) and HFG group (*p*<0.001) was significantly lower than that of mice in the CON group. There was no significant difference between the HF and the HFG groups in food intake (Figure 1(a)). However, after energy correction, dietary genistein significantly reduced the energy intake in the HFG group compared with the HF group (*p*<0.05) and with the CON group (*p*<0.01) (Figure 1(b)). After eight weeks of intervention, the body weight of mice in the HF group was significantly higher than that of mice in the CON group (*p*<0.0001). Dietary genistein significantly reduced the body weight of mice in the HFG group compared with mice in the HF group (*p*<0.0001) and was even lower than the body weight of mice in the CON group (Figure 1(c)).

### 3.2. Genistein Could Counteract the Harmful Effects of a High-Fat Diet on Glucose Tolerance and Insulin Sensitivity

**Figure 2:**
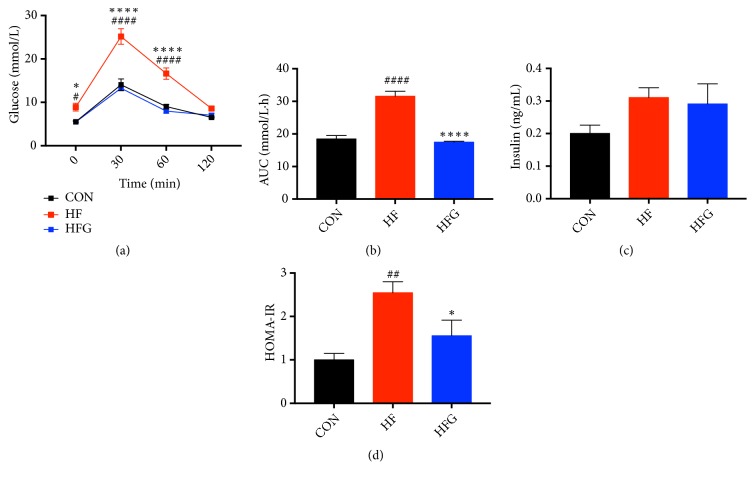
Genistein improved the glucose tolerance and insulin sensitivity in mice fed HFG diet compared with those fed HF diet. (a) IPGTT; (b) AUC; (c) serum insulin levels; (d) HOMA-IR. IPGTT: intraperitoneal glucose tolerance test; AUC: area under the curve; HOMA-IR: the homeostasis model assessment of insulin resistance. CON: normal control diet; HF: high-fat diet without genistein; HFG: high-fat diet with genistein. Data are expressed as means ± S.E.M. (n=8/group). Mean values were significantly different between other groups and the CON group: ^#^*p*<0.05; ^##^*p*<0.01; ^###^*p*<0.001; ^####^*p*<0.0001. Mean values were significantly different between HF group and the HFG group: ^*∗*^*p*<0.05, ^*∗∗*^*p*<0.01, ^*∗∗∗*^*p*<0.001, ^*∗∗∗∗*^*p* <0.0001.

After eight weeks of intervention, mice in the HF group had glucose intolerance as measured by the IPGTT compared with that of mice in the CON group. The blood glucose levels of the HF group were higher at 0 min (*p*<0.05), 30 min (*p*<0.0001), and 60 min (*p*<0.0001), and the AUC was significantly larger for mice in the HF group (*p*<0.0001) than in the CON group. However, dietary genistein could improve the glucose intolerance resulting from the high-fat diet. As shown in Figures 2(a) and 2(b), genistein significantly reduced the blood glucose levels at 0 min (*p*<0.05), 30 min (*p*<0.0001), and 60 min (*p*<0.0001) and the AUC (*p*<0.0001) of mice from the HFG group compared with those of the HF group, even reducing blood glucose to normal levels. Furthermore, to determine the effects of genistein on insulin sensitivity, the level of fasting serum insulin was measured. As shown in Figure 2(c), the serum insulin levels were not significantly different among the three groups. However, the HOMA-IR index was significantly higher in the HF group compared with that of the CON group (*p*<0.01). Dietary genistein could negate the deleterious effects of a high-fat diet on insulin sensitivity and decrease the HOMA-IR index (*p*<0.05) (Figure 2(d)).

### 3.3. Dietary Genistein Could Improve Lipid Metabolism

**Figure 3:**
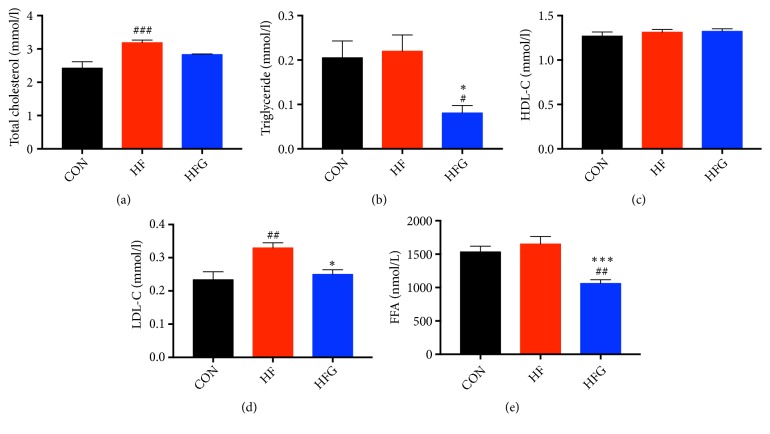
Dietary genistein could improve lipid metabolism. (a) Serum total cholesterol; (b) serum triacylglycerol; (c) serum HDL-C; (d) serum LDL-C, and (e) serum FFA. HDL-C: high-density lipoprotein cholesterol; LDL-C: low-density lipoprotein cholesterol; FFA: free fatty acids; CON: normal control diet; HF: high-fat diet without genistein; HFG: high-fat diet with genistein. Data are expressed as means ± S.E.M. (n=8/group). Mean values were significantly different between other groups and the CON group: ^#^*p*<0.05; ^##^*p*<0.01; ^###^*p*<0.001. Mean values were significantly different between HF group and the HFG group: ^*∗*^*p*<0.05, ^*∗∗*^*p*<0.01, ^*∗∗∗*^*p*<0.001.

To explore the effects of genistein on lipid metabolism, we measured the serum levels of TC, TG, HDL-C, LDL-C, and FFA. The serum TC (*p*<0.001) and LDL-C (*p*<0.01) levels were significantly higher in mice from the HF group than those from mice in the CON group. Dietary genistein significantly reduced the serum levels of TG (p<0.05), LDL-C (*p*<0.05), and FFA (*p*<0.001) in the HFG group compared with those in the HF group (Figures 3(a)–3(e)).

### 3.4. Dietary Genistein Regulates Gene Expression in the Hypothalamus, Especially Genes Related to Circadian Entrainment

**Figure 4:**
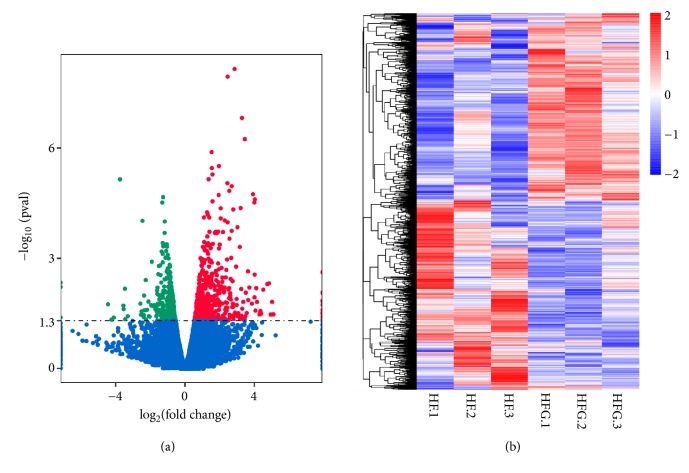
Dietary genistein could regulate gene expression in the hypothalamus. (a) The volcano plot graphs: this graph shows the log2 of the fold change in each gene's expression between the two groups and its -log (*p* value) from the t-test. The red indicates that the gene is upregulated, whereas the green indicates that gene is downregulated; and (b) heatmap diagram: this diagram illustrates the differential expression of hypothalamic mRNAs in the HF group (n = 3) compared with the HFG group (n = 3). The hierarchical clustering was based on 878 differentially expressed mRNAs. HF: high-fat diet without genistein; HFG: high-fat diet with genistein.

The hypothalamus plays a crucial role in the regulation of energy and metabolic homeostasis and circadian rhythm. To explore the central mechanisms of genistein improvement of glycolipid metabolism and regulation of body weight, we determined the gene expression profiles from hypothalamic tissue of mice from the HF group and HFG groups using whole transcriptome sequencing analyses. As shown in Figure 4(a), there were 878 differentially expressed genes identified in the hypothalamic tissues between the two groups, among which 490 genes were upregulated and 388 genes were downregulated in the HFG group compared with the genes of the HF group. Hierarchical clustering of differentially expressed hypothalamic genes also showed significant differences in the transcriptional profiles between the HFG group and those of the HF group (Figure 4(b)). Then, we analyzed the KEGG pathways enriched by the differentially expressed genes in the HFG group.

**Figure 5:**
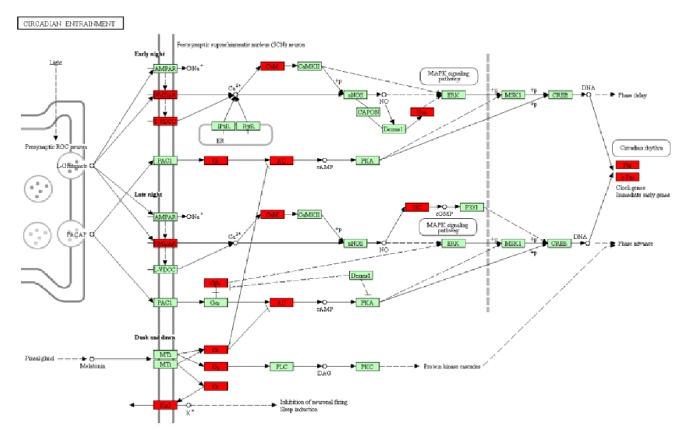
Differentially expressed genes in the HFG group enriched in the KEGG pathway of circadian entrainment. Red represents the different expression genes.

As shown in Table 1, the most abundant pathway was related to the “circadian entrainment” (*p*=0.0016) pathway. The “Rap1 signaling pathway” (*p*=0.0127), “salivary secretion” (*p*=0.0221), “estrogen signaling pathway” (*p*=0.0282), “cGMP-PKG signaling pathway” (*p*=0.0375), “oxytocin signaling pathway” (*p*=0.0417), “cAMP signaling pathway” (*p*=0.0474), and some other pathways were also significantly enriched. In light of the crucial role of circadian rhythm in metabolism, we focused on the “circadian entrainment” pathway. Twelve genes in this pathway were significantly differentially expressed, among which* Per1 *(period circadian clock 1),* c-Fos* (FBJ osteosarcoma oncogene),* Calm1 *(calmodulin 1), and* Gng5 *(G protein subunit gamma 5) were upregulated; the remaining eight genes, including* Grin1 *(glutamate receptor ionotropic),* Cacna1g *(voltage-dependent calcium channel T type alpha-1G),* Kir3.1* (potassium inwardly rectifying channel subfamily J member 3),* Adcy4 *(adenylate cyclase 4),* Gucy1a2 *(guanylate cyclase soluble subunit alpha), and so on were downregulated (Figure 5). In addition to genes in the circadian entrainment pathway,* Cry1* (cryptochrome circadian regulator 1), which is an important clock gene, was also significantly upregulated in mice of the HFG group compared with that in the HF group (*p*<0.05).

### 3.5. Validation of the Whole Transcriptome Analysis of Hypothalamic Gene Expression

**Figure 6:**
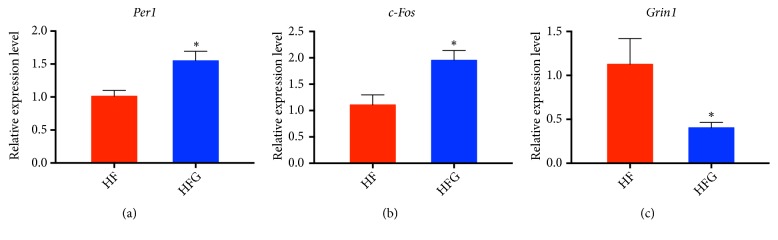
RT-qPCR verification of significant genes between the HF group and the HFG group. (a)* Per1*; (b)* c-Fos*; and (c)* Grin1*. HF: high-fat diet without genistein; HFG: high-fat diet with genistein. Data are expressed as means ± S.E.M. (n=5/group). Mean values were significantly different between HF group and the HFG group: ^*∗*^*p*<0.05.

To verify the sequencing results, we selected three differentially expressed genes,* Per1*,* c-Fos*, and* Grin1*, in the “circadian entrainment” pathway for verification using RT-qPCR. As shown in Figure 6,* Per1 *(*p*<0.05) and* c-Fos *(*p*<0.05) were significantly upregulated in the HFG group, whereas* Grin1 *(*p*<0.05) was downregulated. Thus, a significant consistency was observed between the transcriptome sequencing and the RT-qPCR results, which validated the reliability of our whole transcriptome sequences.

## 4. Discussion

It is well established that the hypothalamus plays a central role in the homeostatic regulation of energy and glucose metabolism as well as in the generation and control of circadian rhythm. Accumulated evidence suggests that hypothalamic central regulation disorder plays an important role in the development of obesity and T2DM [30–33]. Consumption of soy foods and genistein have been reported to be related to a lower risk of T2DM [7]. However, the central mechanism in the hypothalamus for genistein improving glucose and lipid metabolism is still poorly understood. The goal of our study was to investigate the effects of dietary genistein on glucose and lipid metabolism and gene expression in the hypothalamus. We found that dietary genistein could counteract the deleterious effects of a HF diet on glucose homeostasis and the HOMA-IR. Furthermore, dietary genistein significantly reduced body weight compared with mice fed only a HF diet. Disorders of the serum lipid profiles due to the high-fat diet were also prevented after genistein intervention. These changes were accompanied by a reduction in energy intake. These data demonstrated that genistein is vital for regulating body weight and improving glucose and lipid metabolic health, which was in agreement with the previous study that showed that soy isoflavones had beneficial effects on metabolic health [10, 14, 15].

In light of the pivotal role of the hypothalamus in metabolic health, we hypothesized that gene expression in the hypothalamus was altered in mice fed genistein. Indeed, whole transcriptome sequences showed that great changes occurred in gene expression in the hypothalamus after eight weeks of genistein intervention compared with gene expression in the HF group. After enrichment analysis of differentially expressed genes in the HFG group, circadian entrainment was considered the most abundant and crucial pathway. There were twelve significant differentially expressed genes in this pathway, including* c-Fos*,* Grin1*, and* Per1. *And another circadian rhythm-related gene* Cry1* was also significantly upregulated.

The circadian rhythm exists in nearly all living organisms and controls numerous circadian oscillators for physiological processes, including metabolism, wake-sleep cycles, feeding behavior, and body temperature, which have been described as changing in response to the light/dark cycles created by earth's rotation. Multiple metabolic processes, including insulin secretion and energy expenditure, exhibit rhythmic changes over the course of 24 h [34]. In addition to the circadian rhythm in metabolism, growing numbers of clinical studies have indicated that circadian misalignment significantly elevated the levels of glucose, insulin, and TG [21, 22, 35]. Furthermore, animal experiments suggested that mice with* Per* and* Cry* mutations were more susceptible to obesity, impaired glucose tolerance, hyperinsulinemia, and hyperlipidemia [36–38]. Thus, circadian rhythm plays essential roles in metabolic health. Miranda et al. [39] discovered that resveratrol can counteract the altered expression of* Rev-Erbα *(reverse erythroblastosis virus *α*) in adipose tissue induced by a high-fat diet, which indicated that the circadian clock is a potential target for polyphenol. Huang et al. [40] showed that Jiao-Tai-Wan could increase the expression levels of circadian proteins CRY1 and CRY2 in hypothalamus of obesity-resistant rats with chronic partial sleep deprivation, which was associated with improvement in inflammation. The benefits of green tea (-)-epigallocatechin-3-gallate on food intake were confirmed to be associated with the expression of* Clock* and* Bmal1* in hypothalamus of high-fat fed mice [41]. However, research exploring the association between the protective effects of genistein intake against metabolic disorders and the circadian clock is scarce. To our knowledge, this is the first study that demonstrated the beneficial effects of genistein on the role of circadian rhythm, especially the central clock, in metabolism.

The circadian clock contains a central pacemaker that is located in the suprachiasmatic nucleus (SCN) of the hypothalamus and in multiple peripheral clock organs, including the liver, gut, pancreas, kidney, muscle, and adipose tissue. The SCN is entrained primarily by light and is responsible for the integration and coordination of circadian oscillators in the peripheral clocks. In addition, nonphotic behavior cues, including feeding, sleep, and physical activity, all influence the timing of central circadian rhythm [34, 42]. Circadian rhythms are generated and maintained by a series of clock genes, such as* Clock*,* Per1/2*,* Cry1/2*, and* Bmal1,* which form a transcriptional-translational feedback loop. It has been reported that sleep deprivation could reset the central circadian clock and result in significantly decreased expression of C-FOS and PER1 protein, CRY protein, and the* Per1/2* mRNA in the SCN in mice, rats, and hamsters [40, 43, 44]. Maywood et al. [43] also showed that wheel running could modulate the expression of* Per1* and* Per2* in the SCN under constant dark conditions [45]. Furthermore, numerous studies have demonstrated that feeding behavior is one of the major factors to entrain peripheral clocks [46–48]. Although controversy exists, feeding has also been reported to reset the hypothalamic clocks. To explore whether a high-fat diet could influence the SCN clock, Mendoza et al. [49] measured the clock entrainment to light in mice fed a HF diet. They showed that the photic induction of protein expression of C-FOS and P-ERK (phosphorylation of the extracellular signal-regulated kinases I/II) in the SCN were both significantly decreased after consumption of a HF diet. Thus, high-fat feeding could modulate circadian entrainment to light. Another rat study verified that the SCN took part in food-mediated entrainment of circadian rhythm, and, in that study, the expression of C-FOS and PER1 was also regulated in the rats bearing bilateral SCN lesions [50]. In our present study, we found that the addition of dietary genistein resulted in significantly upregulated expression of* c-Fos* and* Grin1 *compared to gene expression in the HF group, which is noteworthy because* Grin1* encodes the glutamatergic receptor-NMDAR protein. Several lines of evidence indicate that glutamate is the main photic signal for the central circadian clock [51, 52].* c-Fos* was also considered a functional marker of light-activated signal transduction pathways in the SCN. Thus, our results indicated that dietary genistein could modulate circadian entrainment in the hypothalamus and negate the deleterious effects of a HF diet on the reduced photic induction of C-FOS. The C-FOS and NMDAR were both key to transmit the photic information to the SCN. The upregulation of these two genes would reset the circadian rhythm and alter the clock synchronization to light, which might further change the rhythm of food intake and metabolism [53]. However, the specific mechanism of the effects of circadian entrainment disorder on peripheral metabolism is still unclear and needs to be further studied.

In addition to circadian entrainment in the SCN, the circadian rhythm in other hypothalamic nuclei was associated with changes in feeding behavior. Kohsaka et al. [54] explored the effects of a HF diet on the behavioral and molecular circadian rhythm in the mediobasal hypothalamus of mice and found that the circadian rhythm of food intake was changed and that the circadian rhythm in the mediobasal hypothalamus was also disrupted in the expression of* Clock* mRNA. Blancas-Velazquez et al. [55, 56] found that a high-fat high-sugar diet could control the expression of* Per2* mRNA and the PER2 and BMAL1 protein in reward-related areas of the hypothalamus rather than the SCN. Feeding only during the daytime in animals was also described to lead to metabolic disorders and disrupted rhythm in the expression of* Per1* and* Per2 *mRNA in the perifornical area and the arcuate nucleus of the hypothalamus [57]. Our results demonstrated that the expression of* Per1* and* Cry1 *was significantly upregulated in the hypothalamus after dietary genistein intervention. However, it is still unclear whether the gene is differentially expressed in the SCN or in the other hypothalamic nuclei. We need to further explore the specific location of the changes in hypothalamic gene expression after genistein intervention in the future.

## 5. Conclusions

Dietary genistein could significantly mitigate the deleterious effects of a HF diet on body weight as well as glucose and lipid metabolism. The improvement in glucose and lipid metabolic disorders was associated with significantly different gene expression in the hypothalamic circadian entrainment pathway. To our knowledge, this is the first study to report the role of hypothalamic circadian entrainment in the improvement of metabolic health and reduction of body weight by dietary genistein intake. However, there are some limitations to our study. First, only female mice were used in our study. Second, our study only explored the relationship between the expression of hypothalamic genes and metabolism, and the specific metabolism-regulating mechanisms of these genes still need further exploration. Lastly, a more accurate nuclear location and understanding of the circadian rhythm of differentially expressed genes in the hypothalamus still need to be determined. Our results provide new evidence for the central regulation of energy and metabolic homeostasis and for novel targets for intervention in obesity and T2DM.

## Figures and Tables

**Table 1:** Kyoto Encyclopedia of Genes and Genomes (KEGG) pathways of differentially expressed genes in the HFG group (*p* < 0.05).

KEGG ID	Pathway Name	O	C	*p* value
04713	Circadian entrainment	12	98	0.0016
04015	Rap1 signaling pathway	17	215	0.0127
04970	Salivary secretion	8	78	0.0221
04915	Estrogen signaling pathway	9	98	0.0282
04640	Hematopoietic cell lineage	8	87	0.0371
04022	cGMP-PKG signaling pathway	13	173	0.0375
04921	Oxytocin signaling pathway	12	158	0.0417
04974	Protein digestion and absorption	8	90	0.0434
04024	cAMP signaling pathway	14	198	0.0474

O: the number of differentially expressed genes in the pathway; C: the number of reference genes in the pathway.

## Data Availability

The data used to support the findings of this study are available from the corresponding author upon request.

## Supplementary Materials

Supplementary Materials Table S1: the nutritional compositions of three types of diet, CON: normal control diet; HF: high-fat diet without genistein; and HFG: high-fat diet with genistein. Table S2: the primers sequences of the four genes,* Per1*: period circadian clock 1;* c-Fos*: FBJ osteosarcoma oncogene;* Grin1*: glutamate receptor ionotropic; and *β*-actin.
