# Supplementary material for: Dietary Genistein Could Modulate Hypothalamic Circadian Entrainment, Reduce Body Weight, and Improve Glucose and Lipid Metabolism in Female Mice
Source: Int J Endocrinol. 2019 Apr 17;2019:2163838. doi: 10.1155/2019/2163838 (PMC6500629; doi:10.1155/2019/2163838)
Supplement: Supplementary Materials — Table S1: the nutritional compositions of three types of diet, CON: normal control diet; HF: high-fat diet without genistein; and HFG: high-fat diet with genistein. Table S2: the primers sequences of the four genes, Per1: period circadian clock 1; c-Fos: FBJ osteosarcoma oncogene; Grin1: glutamate receptor ionotropic; and β-actin. [file 2163838.f1.pdf]

Supplementary material

Table S1. The nutritional compositions of three types of diet. CON, normal control diet; HF, high-fat diet without genistein; HFG, high-fat diet with genistein.

Ingredients	CON(g)	HF(g)	HFG(g)
Casein	200	258	258
L-Cystine	3	4	4
Corn Starch	397	0	0
Maltodextrin	132	162	162
Sucrose	100	89	89
Cellulose	50	65	65
Soybean Oil	0	0	0
Corn Oil	70	32	32
t-Butylhydroquinone	0.014	0	0
Mineral Mix S10026	0	13	13
MinarelMix	35	0	0
Vitamin Mix	0	13	13
Vitamin Mix	10	0	0
Choline Bitartrate	2.5	2.6	2.6
Lard	0	316.6	316.6
DiCalcium	0	16.8	16.8
Calcium Carbonate	0	7	7
Potassium Citrate, 1	0	21	21
FD&C Blue Dye #1	0	0.06	0.06
Genistein	0	0	2
Total	1000	1000	1000

Table S2. The primers sequences of the four genes. *Per1*, period circadian clock 1; *c-Fos*, FBJ osteosarcoma oncogene; *Grin1,* glutamate receptor ionotropic; β-actin.

**Genes**	**Forward**	**Reverse**
Per1	CGGATTGTCTATATTTCGGAG	TGGGCAGTCGAGATGGTGTA
c-Fos	CGGGTTTCAACGCCGACTA	TTGGCACTAGAGACGGACAG
Grin1	AGAGCCCGACCCTAAAAAGAA	CCCTCCTCCCTCTCAATAGC
β-actin	GGCTGTATTCCCCTCCATCG	CCAGTTGGTAACAATGCCAT
